# The Healing Effect of Conditioned Media and Bone Marrow-Derived Stem Cells in Laryngotracheal Stenosis: A Comparison in Experimental Dog Model

**Published:** 2017-05

**Authors:** Kamyar Iravani, Arash Sobhanmanesh, Mohammad Javad Ashraf, Seyed Basir Hashemi, Davood Mehrabani, Shahrokh Zare

**Affiliations:** 1 Otolaryngology Research Center, Department of Otolaryngology, Shiraz University of Medical Sciences, Shiraz, Iran;; 2 Department of Pathology, School of Medicine, Shiraz University of Medical Sciences, Shiraz, Iran;; 3 Stem Cell Technology Research Center, Shiraz University of Medical Sciences, Shiraz, Iran

**Keywords:** Healing, Conditioned media, Bone marrow, Stem cell, Laryngotracheal stenosis, Dog

## Abstract

**BACKGROUND:**

Differences in causes, severities, areas of stenosis, and the association with swallowing and phonation of larynx and trachea can result into Laryngotracheal stenosis (LTS).This study evaluated the healing effect of bone marrow stem cells (BMSCs) in experimentally induced LTS dog model.

**METHODS:**

Seven dogs were enrolled. BMSCs were isolated from proximal humerus and shoulder of a dog and cultured in media containing alpha minimal essential medium, fetal bovine serum, penicillin and streptomycin, and L-glutamine. BMSCs were characterized morphologically, by RT-PCR, and osteogenic induction. Karyotyping was undertaken for chromosomal stability. Mechanical trauma to laryngeal mucosa was identically conducted by Tru-cut punch forceps in right and left vocal folds. Two milliliter of conditioned media or BMSCs (2×10^6^) were injected in the right site of the tissue and the left side was considered as control after LTS induction. The larynx was visualized 2, 4 and 6 weeks after treatment. Six weeks post-treatment, the larynges were evaluated histologically.

**RESULTS:**

BMSCs were adhered to culture flasks, spindle shape and positive for mesenchymal marker and negative for hematopoietic markers. Osteogenic induction was verified by Alizarin red staining. Karyotype was normal. A complete epithelialization and minimal chronic inflammatory cell infiltration were noted in submucosa of both left (control) and right (cases) vocal folds. The healing effect of conditioned media and BMSCs in comparison to the control group was more prominent.

**CONCLUSION:**

As thickness of fibrosis in cases were less than control group, conditioned media and BMSCs were shown to be good choices in healing of LTS.

## INTRODUCTION

The larynx and trachea are semirigid tubular organs when in the tissue, concentric scar contraction, a normal wound healing process happen may lead to narrowing of the lumen. Differences in causes, severities, areas of stenosis, and the association with swallowing and phonation can result into Laryngotracheal stenosis (LTS).^1^ Causes for LTS include external trauma, post-intubation injury, and iatrogenic injuries.^2^^,^^3^ Traumatic cases present various problems due to the unpredictability of pattern and extent of trauma. Additional injuries may complicate it including vocal fold paralysis, mandibular fractures compromising the upper airway, chest injuries compromising the lower airways, and other pre-existing co-morbidities.^4^^,^^5^

The extracellular matrix (ECM) as a biological scaffold derived from decellularized organs or tissues with no immunogenicity was shown to provide a perfect three-dimensional architecture for the tissue regeneration and induces adherence and proliferation of cells.^6^ Attempts to construct an entire bioartificial larynx have involved several approaches because the larynx is a complicated organ comprising multiple tissues. The laryngeal framework is principally composed of thyroid cartilage, cricoids cartilage, and arytenoid cartilage. Posterior cricoarytenoid muscle, lateral cricoarytenoid muscle, thyroarytenoid muscle, and vocal folds are inserted in the framework. It was proved that cartilage was an organ with low immunity, and therefore larynx immunogens are positioned mainly in the mucosa and muscles.^7^

Regenerative medicine has opened a new window in treatment cartilage defects applying mesenchymal stem cells (MSCs) based on their multilineage differentiation property to cartilage tissue.^8^ MSCs have immunomodulatory and anti-inflammatory potential, self-renewal capacity, stemness maintenance and plasticity.^9^ MSCs were isolated from different tissues including bone marrow,^10^ adipose tissue,^11^ and menstrual blood.^12^ They have osteogenic,^13^ adipogenic,^14^ and neurogenic^15^ differentiation properties. Dogs are valuable experimental model for the study of new therapiesforhumandiseases.^16^ Yet, no approved therapeutic measure has been introduced for reconstruction of LTS using MSCs in a canine model. This study was undertaken to evaluate the healing effect of bone marrow stem cells (BMSCs) in experimentally induced LTS in dog model.

## MATERIALS AND METHODS

Seven dogs (1 y/o, male, mixed breed) were provided from Laboratory Animal Center of Shiraz University of Medical Sciences, Shiraz, southern Iran. During the experiments, the animals were housed in standard cages as one per cage at a temperature of 20±2ºC and humidity of 55±5% using a 12 h light and 12 h dark cycle and could move freely. Standard food and water were available ad libitum. All dogs were treated in compliance with the recommendations of the Animal Care Committee of the Shiraz University of Medical Sciences. This study was approved in institution ethics committee too.

To isolate BMSCs, the upper part of proximal humerus and shoulder of a dog were shaved under general anesthesia using diazepam (0.2 mg/kg, Darou Pakhsh, Iran) and ketamine (10 mg/kg, Alfasan, Netherlands). The animals were positioned in lateral recumbency to get bone marrow from the proximal humerus, and the shoulder joint. Jamshidi needle comprising 1-ml heparin (5000 IU/ml, Alborz Darou, Iran) was used to collect bone marrow which was later maintained in the ice.

An equal volume of Dulbecco’s Modified Eagle’s Medium (DMEM, Biovet, Bulgaria) was added to the aspirated bone marrow; over an equal volume of Ficoll-Paque product (GE Healthcare Life Sciences, UK, without intermixing) that was layered carefully. The sample was centrifuged at 1500 rpm for 30 min at 20°C. The interface between the plasma and the Ficoll-Paque layer contained mononuclear cells (MNCs) that was separated and cultured in 10% fetal bovine serum (FBS, Biovet, Bulgaria), 88% alpha minimal essential medium (αMEM, Biovet, Bulgaria), 1% penicillin and streptomycin, and 1% L-glutamine (Sigma cat. no. G5840, the OCED and the EU) in 75-cm^2^ culture flasks. 

MNCs were expandedin an incubator at 37°C with saturated humidity and 5% CO2. The media was first changed after 24 h while non-adherent hematopoietic cells were removed. Then the culture medium was replaced every 3–4 days. To subculture cells at confluency of 80–90%, the adherent cells were washed twice with PBS, and harvested using 0.25 % trypsin (Gibco, USA) for 3 min. The enzyme was inactivated with an equal amount of culture media. Canine MSCs were passaged up to passage 3 and at each passage, the cells were morphologically evaluated.

To characterize canine BMSCs by RT-PCR, total RNA was extracted using RNX-Plus buffer (Cinnagen, Iran), as described before.^10^ RNA quantification and integrity assessment, cDNA synthesize from DNA-free RNA and designing gene-specific primers for CD90, CD45, and CD34, RT-PCR were conducted as previously reported.^10^ The products were visualized under UV light.

For in vitro osteogenic differentiation of BMSCs, cells at 90% confluence were expanded in DMEM, 15% FBS, 200 µM L-ascorbic acid, 10 mM glycerol phosphate and 100 nM dexamethasone. The medium was replaced two times per week for 3 weeks. After 3 weeks, osteogenic differentiation was evaluated using alizarin red staining that was bound to calcium ions in mineralized deposits leading to a brilliant red staining (Sigma-Aldrich, USA). For karyotyping analysis of BMSCs, they were harvested when reached 70% confluence. One-hundred μl colcemide was added in 25-cm^2^ tissue culture flasks for 30, 45, and 60 min to passage 4 of BMSCs. Then for 45 and 75 min to passage 4 of the cells and, the third time for 1.5, 3, and 5.5 h to passage 5 of the cells, and finally for 10 h to passages 1 and 8, and incubated at 37°C. The top mediums were removed and rinsed twice with phosphate buffered saline (PBS). Cells were removed from the flask using 1.5 ml trypsin. 

To inactivate the enzyme, 3 ml of culture media containing FBS was added and later was centrifuged for 7 min at 1200 rpm. The cells were later lysed in hypotonic solution using 0.075 M KCl and were incubated for 20 min in a 37°C water bath. Then, fixation in 1-ml methanol/acetic acid (3:1) was carried out and incubated for 10 min in a 37°C water bath. The suspension was centrifuged for 6 min at 1200 rpm. The upper medium was removed and fixed in 3-ml methanol/acetic acid (3:1). Again, the suspension was centrifuged for 6 min at 1200 rpm, and the uppermediumwas poured off. This step was repeated three times. 

The cell suspension was kept in methanol/acetic acid at −20 °C. Three drops of the suspensionwere transferred to one side of the slide and then, the slide was tilted to permit the suspension to run down to the other end of the slide to prepare spreading of metaphase chromosomes. They were stained with Giemsa and the chromosomes were counted. Image analysis of metaphase chromosome spreads were acquired using a Nikon microscope Eclipse E600 (Nikon Instruments Inc., Melville, NY) and Cytovision software version 3.1 (Applied Imaging Corp., USA). The chromosome numbers per spread were counted for 20 spreads of the early and late passages.

To undergo surgery, all 7 animals were anesthetized with sodium pentobarbital (30 mg/kg). Larygoscope was introduced into the mouth and throat.Larynx was visualized using a microscope. Mechanical trauma to the laryngeal mucosa was conducted by a Tru-cut punch forceps for all tissuesanda defect was created in the right and left vocal folds. All defects were equal in size and in the true vocal cords. For treating with BMSCs conditioned media, the supernatant of the BMSCs culture flasks lacking any cells was provided as 2 mL conditioned media. The provided conditioned media was injected in the right site of the tissue and the left side was considered as control without any treatment intervention after induction of LTS in all dogs. MSCs were transplanted in the right side (2×10^6^ in 2 ml of media) while the left side similar to the condition media group was considered as control in all animals. To prevent infection, 3,000,000 units of streptomycin were administered daily for the first 3 days after the operation. 

To assess the healing, the larynx was observed by macroscopic visualization 2, 4 and 6 weeks after treatment. Six weeks after treatment with conditioned media and BMSCs, the animals were euthanized by an overdose injection of the anesthetic drug and the larynges were removed and transferred into 10% buffered formalin for histological evaluation. In gross examination, the site of injury and subsequent repair were assessed. Then tissue sections for microscopic examination were taken from symmetrical right and left true vocal folds. Tissue sections were cut and stained by Hematoxillin & Eosin (H&E) and also by Masson Trichrome staining. 

Histologic slides were visualized for changes in epithelialization and degree and type of submucosal inflammation using light microscope (Olympus model BX 53). Inflammation was graded as severe, moderate, mild and minimal according to density of inflammatory cells. The thickness of submucosal fibrosis was photographed and measured in micrometer in Masson Trichrome staining (Olympus camera model DP 70) based on its software. Histological data was compared in both sides. 

## RESULTS

**Fig. 1:**
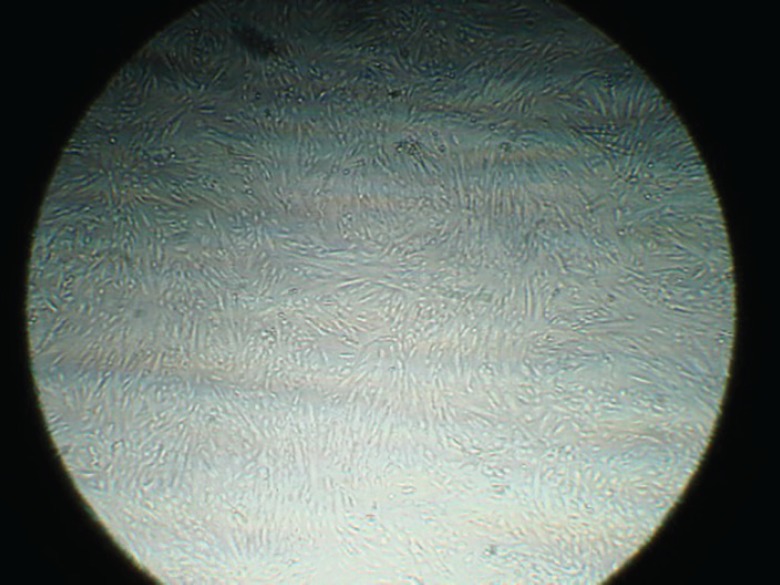
BMSCs are seen as spindle shape morphology in passage 3 (×20

**Fig. 2:**
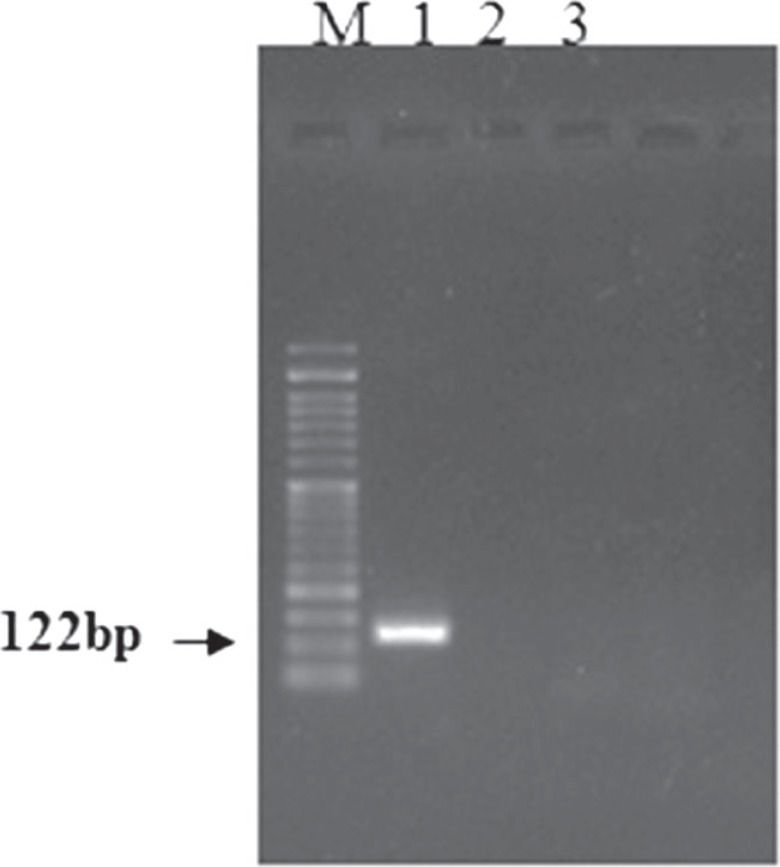
RT-PCR of BMSCs were positive for CD90 and negative for CD34 and CD45 (M, 50 bp ladder; lane1, CD90; lane2, CD34; lane3, CD45

**Fig. 3:**
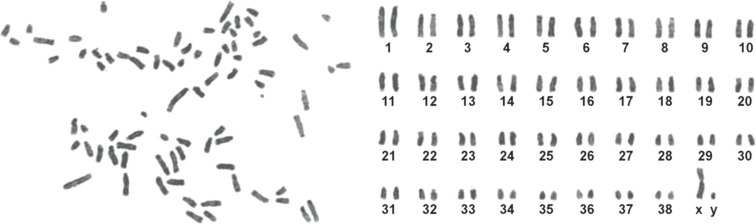
Canine chromosomes at metaphase (left) and karyotyping (right) of BMSCs (2n=78) were shown

**Fig. 4:**
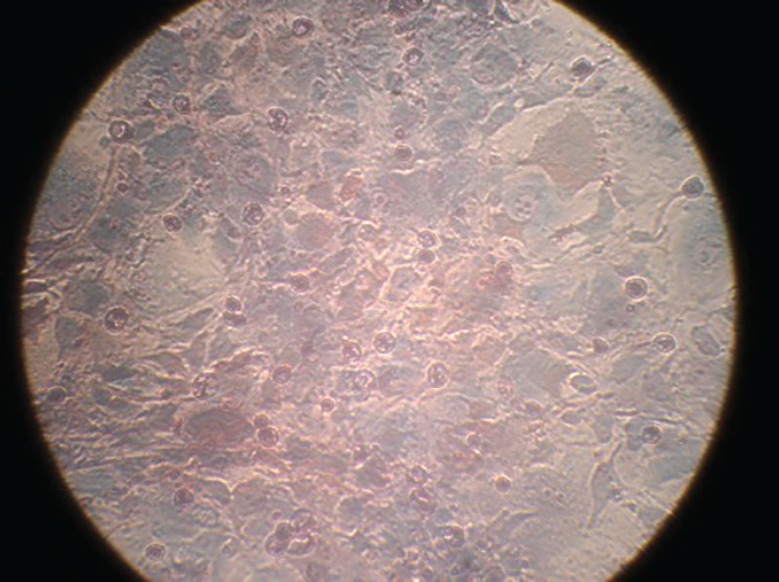
Alizarin red staining denoted to calcification and osteogenic differentiation of BMSCs

The isolated BMSCs were adhered to culture flasks after 24 h. As shown in Figure 1, fibroblast-like cells to be spindle shape in morphology were visible up to passage 4. BMSCs were positive for mesenchymal marker of CD90 and negative for hematopoietic markers of CD34 and CD45 using RT-PCR (Figure 2). Karyotype analysis of BM-MSCs at passage 3 was normal with diploid number of chromosomes (2n=78; Figure 3) at passage 3 (76 autosomes of 38 pairs and 2 sex chromosomes of 78XX or 78XY). Autosomes were acrocentric while both sex chromosomes were bi-armed. The X chromosome was a large metacentric chromosome, and the Y chromosome was a small metacentric chromosome. After 21 days of plating of BMSCs in osteogenic media, osteogenic differentiation was noticed by presence of calcium deposits when stained by Alizarin red (Figure 4).

**Fig. 5:**
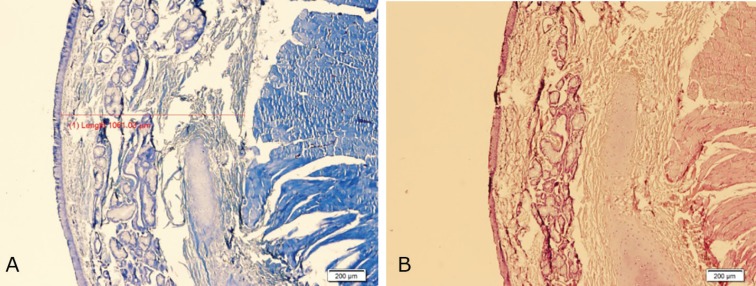
Complete epithelialization and minimal chronic inflammatory cell infiltration in left vocal fold (Control) sections with significant amount of submucosal fibrosis (**A: **H&E, **B:** Masson Trichrome, ×40

**Fig. 6:**
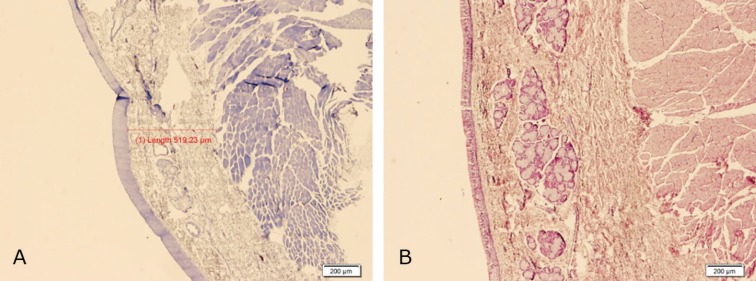
Complete epithelialization and minimal chronic inflammatory cell infiltration and relatively less submucosal fibrosis in right vocal fold (Case) sections (**A:** H&E, **B:** Masson Trichrome, ×40

Histological examinations revealed a complete epithelialization and minimal chronic inflammatory cell infiltration in submucosa of both left (control) and right (cases) vocal folds (Figure 5 and 6). The findings denoted to the healing effect of conditioned media and BMSCs in comparison to the control group although the differences were not significant. In our study, there was no difference in epithelialization, degree and type of inflammation between control and case groups. The average thickness of submucosal fibrosis in the right side (conditioned media) was 764.5 and for BMSCs was 913.8 µm. These figures for the left (control) sides of larynges in conditioned media and BMSCs were 1004.5 and 965.2 µm, respectively. In 57.1% of BMSC cases, the thickness of fibrosis in right sides of larynx were less than the control group. Fibrosis was less in BMSC group (42.9%) than the conditioned media group (50%), but the difference was not statistically significant.

## DISCUSSION

There are few reports on healing effect of conditioned media in tissue defects. The protective and healing effect of conditioned medium from BMSCs have been studied in acute myocardial infarction, confirming the role of MSCs in turnover dynamicsof tissue injury similar to our findings.^17^ In our study, the healing effect of BMSCs conditioned media was evaluated in LTS that is in agreement with other previously reports in othertissue using MSCs in treatment of tissue damages such as heart,^17^^,^^18^ lung,^19^ kidney,^20^ and liver.^21^ The MSCs were shown to secrete several bioactive factors that can provide a microenvironment helping the rearrangement of damaged tissues.^20^

In our study, there was no difference in epithelialization, degree and type of inflammation between control and case groups. In 57.1% of BMSC cases, the thickness of fibrosis in right sides of larynx were less than the control group. Fibrosis was less in BMSC group (42.9%) than the conditioned media group (50%), but the difference was not statistically significant. No statistically significant difference was noted between 2 groups that may be due to low number of subjects, but a trend can be seen that may be considered as effectiveness of BMSCs in comparison to the conditioned media group in reducing fibrosis following vocal fold repair after mechanical injury.

Mankarious *et al. *studied specimens resected from the subglottis and larynx from six patients who had undergone stenosis resection and primary anastomosis. Using immunohistochemical methods, the authors analyzed the specimens for changes in the principle components of hyaline cartilage, collagen types I and II, and the principle proteoglycan secreted by chondrocyte, aggrecan. Normal tracheal and cricoid cartilage contains high ratios of collagen type I to type II compared with articular hyaline cartilage, but the investigators found that in specimens’ cartilage fracture sites, the relative amounts of collagen type I and aggrecan are decreased. In areas of injury demonstrating regenerative cartilage growth, amounts of collagen type II and aggrecan were greatly increased.^22^

These results were consistent across a wide range of patient ages. These findings suggest that collagen type I and aggrecan are responsible for the structural integrity of tracheal and cricoid rings, while regenerative fibroblasts do not deposit collagen type I, the main component in normal ring cartilage.^22^ Hou *et al.* designed a study with the aim of recellularization of the decellularized laryngeal muscle with MSCs, and constructed a low-immune heterogenic laryngeal graft in rabbits. Finally, they concluded that reseeding of MSCs into the decellularized laryngeal muscle matrix for construction of a tissue-engineered larynx is feasible.^23^

The indications for LTS reconstruction are numerous including obstructing tracheal tumors, trauma, post-intubation, and tissue reactions. Although in the past years much progress has been made, none of the new developed techniques have resulted in large-scale clinical application. The use of prosthetic materials, stents, tissue flaps, auto grafts, or a combination of these methods have been reported, but complications include migration, dislodgement, material degradation/failure, bacterial chronic infection, obstruction because of granulation tissue, stenosis, necrosis, anastomosis failure, erosion of major blood vessels, life-long immunosuppression, lack of appropriate donor source, lack of adequate vascularization, and lack of epithelium.^24^

LTS is often a combination of cartilage and mucosal abnormalities. Intact epithelial line is of great importance since it prevents the ingrowth of granulation tissue, which leads to fatal airway obstruction. If the epithelium fails to cover the granulation tissue, the growth of granulation tissue becomes profuse and obstructs the tracheal lumen. After weeks or months, the granulation tissue becomes an avascular scar, containing only a few blood vessels.^22^ MSCs possess high proliferative and multipotent differentiation capacities. They can differentiate into every tissue of the mesoderm (e.g. chondrocytes, myocytes, vascular endotheliocytes). They have therefore, a great role in making a ‘‘regenerative environment’’.^25^

Besides working as a cellular source for regeneration, MSCs can secrete various growth factors, cytokines, chemokines, and even produce ECM, resulting in powerful immunemodulative and paracrine regenerative effects. The paracrine effect of the MSCs might build the regenerative tissue microenvironment, that secreted in BMSCs conditioned media too, eventually leading to tissue recovery.^26^^,^^27^ There are few reports on healing effect of BMSCs conditioned media in tissue defects. This study represents experiment designed to investigate regenerative effects of BMSCs and BMSCs conditioned media in induced LTS caused by mechanical injury. We hypothetized that the application of BMSCs and BMSCs conditioned media might be able to heal induced LTS. 

Verwoerd* et al. *also indicated perichondrial injury in 4-week-old rabbits caused disruption and reserved the curvature of the cricoid cartilage ring.^28^ *Mankarious et al. *revealed that cartilagenous injury in rabbit subglottis developed marked fibrosis in cricoid cartilage that ingrowth of fibrotic tissue may cause intraluminal narrowing.^22^ Macchiarini *et al. *prepared a bioartificial patch starting from muscle cell and fibroblasts isolated from a biopsy obtained from the patient (i.e., the future recipient of the transplant). These cells were seeded onto a collagen network obtained from a decellularized porcine jejunal segment (the porcine tissue was gradually replaced by autologous connective tissue.^29^

We present these data as an observational study that needs to be confirmed with larger numbers of cases and additional studies to establish the mechanism behind these findings. As the thickness of fibrosis in case groups were less than the control groups, conditioned media from BMSCs and BMSCs were shown to be good choices in healing of LTS, even the supporting data for BMSCs were more prominent that can be added to the literature for reconstruction of laryngotracheal defects.
